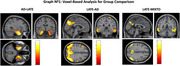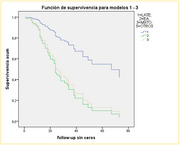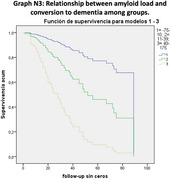# LATE‐like Hypometabolism Pattern in a Cohort of Patients with Mild Cognitive Impairment: Clinical Features, Amyloid Burden, and Conversion to Dementia

**DOI:** 10.1002/alz70856_102056

**Published:** 2025-12-25

**Authors:** Rafael Angel Villino‐Rodríguez, Mario Riverol, Fernando Minguez, Elena Prieto, Christian Espinoza‐Vinces, Cristina Pérez‐Prol, Ainhoa Atorrasagasti‐Villar, Adolfo Jimenez‐Huete, Javier Arbizu

**Affiliations:** ^1^ University Clinica of Navarra, Pamplona, Navarra, Spain; ^2^ University Clinic of Navarra, Pamplona, navarra, Spain; ^3^ University Clinic of Navarra, Pamplona, Navarra, Spain

## Abstract

**Background:**

To describe the incidence of medial temporal hypometabolism as a pattern suggestive of LATE in patients with MCI, along with clinical characteristics, amyloid burden, and conversion to AD dementia.

**Method:**

We studied 207 MCI patients (mean age 72±6.1; MMSE 27±2.6; 50.8% male) who underwent FDG‐PET and amyloid PET imaging, with follow‐up over 24±17.5 months. FDG‐PET patterns were classified as LATE‐like (medial temporal), AD, and mixed (AD‐LATE). Differences among these patterns were analyzed using SPM12, comparing to healthy controls. Amyloid PET was classified visually and quantified using the Centiloid scale. Statistical tests (Chi‐square, ANOVA, and Cox regression) were applied to assess clinical features, amyloid burden, and AD dementia conversion rates.

**Result:**

LATE‐like patterns were identified in 22 patients (10.6%), AD patterns in 57 (27.5%), and mixed patterns in 21 (10.1%). The remaining 107 patients (51.7%) with non‐AD patterns were excluded. The LATE‐like group showed prominent medial temporal hypometabolism, while posterior cingulate and parietotemporal hypometabolism were predominant in AD and mixed groups. LATE‐like patients had higher MMSE scores, lower amyloid positivity, and lower Centiloid values compared to AD and mixed groups. Conversion to AD dementia was slower in LATE‐like patients compared to AD (*p* = 0.005; HR=0.26 [0.1‐0.67]) and mixed (*p* = 0.02; HR=0.29 [0.11‐0.83]).

**Conclusion:**

The LATE‐like FDG‐PET pattern is not uncommon in MCI. It is linked to better cognitive performance, lower amyloid burden, and slower progression to AD dementia compared to AD and mixed patterns.